# Supplementary material for: Emergence of large-scale cell death through ferroptotic trigger waves
Source: Nature. 2024 Jul 10;631(8021):654–62. doi: 10.1038/s41586-024-07623-6 (PMC11639682; doi:10.1038/s41586-024-07623-6)
Supplement: Supplementary file 1 — Uncropped blot for Extended Data Fig. 8b. [file 41586_2024_7623_MOESM1_ESM.pdf]

nature portfolio

https://doi.org/10.1038/s41586-024-07623-6

Supplementary information

Emergence of large-scale cell death through ferroptotic trigger waves

In the format provided by the authors and unedited

Emergence of large-scale cell death via trigger waves of ferroptosis

Hannah K. C. Co, Chia-Chou Wu, Yi-Chen Lee, Sheng-hong Chen*

* **Correspondence**: shengchen@gate.sinica.edu.tw

Supplementary Information

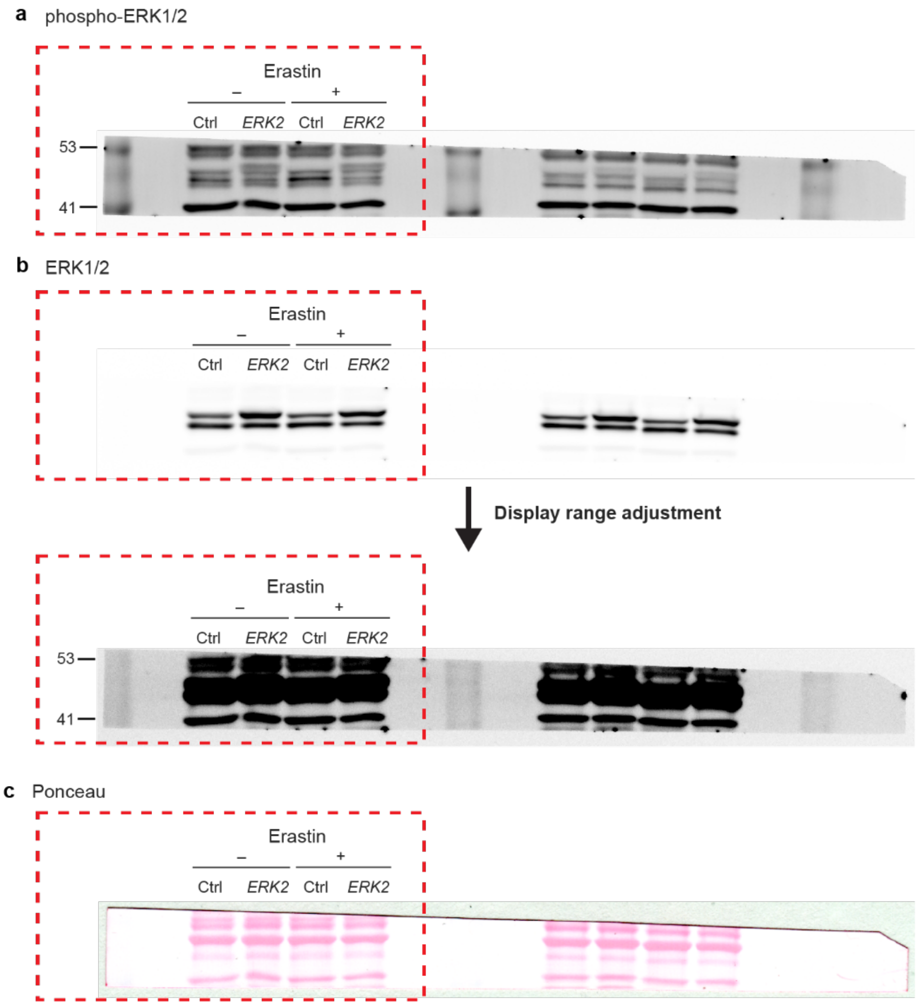

Supplementary Figure 1. Uncropped blot for Extended Data Fig. 8b.

a, **b**, Western blots of phospho-ERK1/2 (**a**) and ERK1/2 with the molecular markers (**b**). The red box indicates the cropped region shown in Extended Data Fig. 8b. **c**, Ponceau staining of blot in (**a**, **b**). (**a**-**c**) are the same blot.
