# Supplementary material for: Emergence of large-scale cell death through ferroptotic trigger waves
Source: Nature. 2024 Jul 10;631(8021):654–62. doi: 10.1038/s41586-024-07623-6 (PMC11639682; doi:10.1038/s41586-024-07623-6)
Supplement: Supplementary file 2 — Reporting Summary [file 41586_2024_7623_MOESM2_ESM.pdf]

nature portfolio

Corresponding author(s): Sheng-hong Chen

Last updated by author(s): May 17, 2024

Reporting Summary

Nature Portfolio wishes to improve the reproducibility of the work that we publish. This form provides structure for consistency and transparency in reporting. For further information on Nature Portfolio policies, see our Editorial Policies and the Editorial Policy Checklist.

Please do not complete any field with "not applicable" or n/a. Refer to the help text for what text to use if an item is not relevant to your study.
For final submission: please carefully check your responses for accuracy; you will not be able to make changes later.

Statistics

For all statistical analyses, confirm that the following items are present in the figure legend, table legend, main text, or Methods section.

n/a	Confirmed	
☐	☒	The exact sample size (*n*) for each experimental group/condition, given as a discrete number and unit of measurement
☐	☒	A statement on whether measurements were taken from distinct samples or whether the same sample was measured repeatedly
☐	☒	The statistical test(s) used AND whether they are one- or two-sided<br>*Only common tests should be described solely by name; describe more complex techniques in the Methods section.*
☒	☐	A description of all covariates tested
☐	☒	A description of any assumptions or corrections, such as tests of normality and adjustment for multiple comparisons
☐	☒	A full description of the statistical parameters including central tendency (e.g. means) or other basic estimates (e.g. regression coefficient) AND variation (e.g. standard deviation) or associated estimates of uncertainty (e.g. confidence intervals)
☐	☒	For null hypothesis testing, the test statistic (e.g. F, t, r) with confidence intervals, effect sizes, degrees of freedom and P value noted<br>*Give P values as exact values whenever suitable.*
☒	☐	For Bayesian analysis, information on the choice of priors and Markov chain Monte Carlo settings
☒	☐	For hierarchical and complex designs, identification of the appropriate level for tests and full reporting of outcomes
☒	☐	Estimates of effect sizes (e.g. Cohen's *d*, Pearson's *r*), indicating how they were calculated

Our web collection on statistics for biologists contains articles on many of the points above.

Software and code

Policy information about availability of computer code

Data collection	Imaging data were collected with Zeiss LSM980 confocal microscope using Zen Blue software (version 3.8), and Zeiss Axio Observer 7 inverted microscope using Zen Blue software (version 3.1). Quantitative PCR was performed on a Bio-Rad CFX 96 with CFX Maestro software 2.3 (version 5.3.022.1030). Mathematical simulations were performed with MATLAB (version R2023b). Custom codes are deposited in GitHub: https://github.com/imb-lcd/ftw2024
Data analysis	Image processing and data analyses were performed in MATLAB (version R2023b), ImageJ (version 1.54f), and Bitplane Imaris (version 10.0.1).

For manuscripts utilizing custom algorithms or software that are central to the research but not yet described in published literature, software must be made available to editors and reviewers. We strongly encourage code deposition in a community repository (e.g. GitHub). See the Nature Portfolio guidelines for submitting code & software for further information.

Data

Policy information about availability of data

All manuscripts must include a data availability statement. This statement should provide the following information, where applicable:

- Accession codes, unique identifiers, or web links for publicly available datasets
- A description of any restrictions on data availability
- For clinical datasets or third party data, please ensure that the statement adheres to our policy

All data are available within the Article and Supplementary Information. Microscopy data are deposited in Figshare (https://doi.org/10.6084/

m9.figshare.25762806). Additional supporting microscopy data are available from the corresponding author upon request, without any restrictions. Source data are provided with this paper.

Research involving human participants, their data, or biological material

Policy information about studies with human participants or human data. See also policy information about sex, gender (identity/presentation), and sexual orientation and race, ethnicity and racism.

Reporting on sex and gender	This study did not involve human participants.
Reporting on race, ethnicity, or other socially relevant groupings	This study did not involve human participants.
Population characteristics	This study did not involve human participants.
Recruitment	This study did not involve human participants.
Ethics oversight	This study did not involve human participants.

Note that full information on the approval of the study protocol must also be provided in the manuscript.

Field-specific reporting

Please select the one below that is the best fit for your research. If you are not sure, read the appropriate sections before making your selection.

☒ Life sciences ☐ Behavioural & social sciences ☐ Ecological, evolutionary & environmental sciences

For a reference copy of the document with all sections, see nature.com/documents/nr-reporting-summary-flat.pdf

Life sciences study design

All studies must disclose on these points even when the disclosure is negative.

Sample size	No statistical method was used to predetermine sample size. Samples sizes were determined based on previous studies with similar methodologies: Chang, J. & Ferrell Jr, J., Nature, 2013; Cheng, X. & Ferrell Jr, J., Science, 2018; Huang, J. et al., Molecular Systems Biology, 2021; Cordeiro, I. et al., Developmental Cell, 2019.
Data exclusions	No data exclusions.
Replication	All experimental results were validated by at least three independent experiments. All experimental findings are reproducible.
Randomization	For cell culture experiments, samples were randomized when possible. For the animal experiment in Fig. 5f, the left and right limbs of an individual animal were randomly allocated in control and experimental groups. For Fig. 5j and Extended Data Fig. 12h and 12i, animals were randomly allocated in control and experimental groups.
Blinding	Investigators were not blinded during data collection. However, data quantification was performed automatically using computational algorithms as described in Methods.

Reporting for specific materials, systems and methods

We require information from authors about some types of materials, experimental systems and methods used in many studies. Here, indicate whether each material, system or method listed is relevant to your study. If you are not sure if a list item applies to your research, read the appropriate section before selecting a response.

Materials & experimental systems

n/a	Involved in the study	
☐	☒	Antibodies
☐	☒	Eukaryotic cell lines
☒	☐	Palaeontology and archaeology
☐	☒	Animals and other organisms
☒	☐	Clinical data
☒	☐	Dual use research of concern
☒	☐	Plants

Methods

n/a	Involved in the study	
☒	☐	ChIP-seq
☒	☐	Flow cytometry
☒	☐	MRI-based neuroimaging

Antibodies

Antibodies used	Primary antibodies used were Erk (1:2000 for WB, Cell Signaling Technology, #9102), phospho-Erk (1:1000 for WB, Cell Signaling Technology, #9106S), 4-HNE (1:250 for IF, Abcam, ab46545 and 1:250 for IF, Abcam, ab48506), and myosin heavy chain (2 μg/mL for IF, Developmental Studies Hybridoma Bank, MF20). Secondary antibodies used were Goat anti-Rabbit IgG Alexa Fluor Plus 488 (1:500, Invitrogen, A32731), Goat anti-Mouse IgG Alexa Fluor Plus 647 (1:500, Invitrogen, A32728), and Goat anti-Rabbit IgG Alexa Fluor 568 (1:500, Invitrogen, A11036).
Validation	Erk antibody (Cell Signaling Technology, #9102) and phospho-Erk antibody (Cell Signaling Technology, #9106) were validated for WB in human cells on manufacturer's website (https://www.cellsignal.com/products/primary-antibodies/p44-42-mapk-erk1-2-antibody/9102 & https://www.cellsignal.com/products/primary-antibodies/phospho-p44-42-mapk-erk1-2-thr202-tyr204-e10-mouse-mab/9106, respectively).<br>4-HNE antibody (Abcam, ab46545) is species independent and was validated for IF in a previous publication (Feng, H. et al., Cell Reports, 2020).<br>4-HNE antibody (Abcam, ab48506) is species independent and was validated for IF on manufacturer's website (https://www.abcam.com/products/primary-antibodies/4-hydroxynonenal-antibody-hnej-2-ab48506.html).<br>Myosin heavy chain antibody (Developmental Studies Hybridoma Bank, MF20) was used in avian tissues and validated for IF in a previous publication (Bader, D. et al., Journal of Cell Biology, 1982).<br>Secondary antibodies Goat anti-Rabbit IgG Alexa Fluor Plus 488 (1:500, Invitrogen, A32731), Goat anti-Mouse IgG Alexa Fluor Plus 647 (1:500, Invitrogen, A32728), and Goat anti-Rabbit IgG Alexa Fluor 568 (1:500, Invitrogen, A11036) were validated for IF based on manufacturer's website (https://www.thermofisher.com/antibody/product/Goat-anti-Rabbit-IgG-H-L-Highly-Cross-Adsorbed-Secondary-Antibody-Polyclonal/A32731; https://www.thermofisher.com/antibody/product/Goat-anti-Mouse-IgG-H-L-Highly-Cross-Adsorbed-Secondary-Antibody-Polyclonal/A32728; https://www.thermofisher.com/antibody/product/Goat-anti-Rabbit-IgG-H-L-Highly-Cross-Adsorbed-Secondary-Antibody-Polyclonal/A-11036).

Eukaryotic cell lines

Policy information about cell lines and Sex and Gender in Research

Cell line source(s)	hTERT RPE-1 (CRL-4000), 786-O (CRL-1932), G-402 (CRL-1440), HOS (CRL-1543), LN-18 (CRL-2610), U-118 MG (HTB-15), PANC1 (CRL-1469), MDA-MB-231 (HTB-26), HT-1080 (CCL-121), NCI-H1650 (CRL-5883), A549 (CRM-CCL-185), U-2 OS (HTB-96), A-172 (CRL-1620), Hs 895.T (CRL-7637), HeLa (CCL-2), and SH-SY5Y (CRL-2266) were obtained from ATCC. HuH-7 (JCRB0403) was obtained from JCRB Cell Bank.
Authentication	None of the cell lines used were authenticated.
Mycoplasma contamination	All cell lines were tested negative for mycoplasma contamination.
Commonly misidentified lines<br>(See ICLAC register)	No commonly misidentified cell lines were used.

Animals and other research organisms

Policy information about studies involving animals; ARRIVE guidelines recommended for reporting animal research, and Sex and Gender in Research

Laboratory animals	Fertilized Leghorn chicken eggs (stage HH30-33) were used in this study.
Wild animals	This study did not involve wild animals.
Reporting on sex	Sex-based analysis was not performed.
Field-collected samples	This study did not involve samples collected from the field.
Ethics oversight	No ethical approval was required for experiments using chick embryos at embryonic stages HH30-HH33.

Note that full information on the approval of the study protocol must also be provided in the manuscript.

Plants

Seed stocks	*Report on the source of all seed stocks or other plant material used. If applicable, state the seed stock centre and catalogue number. If plant specimens were collected from the field, describe the collection location, date and sampling procedures.*
Novel plant genotypes	*Describe the methods by which all novel plant genotypes were produced. This includes those generated by transgenic approaches, gene editing, chemical/radiation-based mutagenesis and hybridization. For transgenic lines, describe the transformation method, the number of independent lines analyzed and the generation upon which experiments were performed. For gene-edited lines, describe the editor used, the endogenous sequence targeted for editing, the targeting guide RNA sequence (if applicable) and how the editor was applied.*
Authentication	*Describe any authentication procedures for each seed stock used or novel genotype generated. Describe any experiments used to assess the effect of a mutation and, where applicable, how potential secondary effects (e.g. second site T-DNA insertions, mosiacism, off-target gene editing) were examined.*

This checklist template is licensed under a Creative Commons Attribution 4.0 International License, which permits use, sharing, adaptation, distribution and reproduction in any medium or format, as long as you give appropriate credit to the original author(s) and the source, provide a link to the Creative Commons license, and indicate if changes were made. The images or other third party material in this article are included in the article's Creative Commons license, unless indicated otherwise in a credit line to the material. If material is not included in the article's Creative Commons license and your intended use is not permitted by statutory regulation or exceeds the permitted use, you will need to obtain permission directly from the copyright holder. To view a copy of this license, visit http://creativecommons.org/licenses/by/4.0/
